# Ceramides may Play a Central Role in the Pathogenesis of Alzheimer’s Disease: a Review of Evidence and Horizons for Discovery

**DOI:** 10.1007/s12035-025-04989-0

**Published:** 2025-04-28

**Authors:** Hans O. Kalkman, Lukasz Smigielski

**Affiliations:** Child and Adolescent Psychiatry and Psychotherapy, Psychiatric University Hospital Zurich, University of Zurich, Zurich, Switzerland

**Keywords:** De novo ceramide synthesis, Sphingomyelinase, Saturated fatty acids, Mitochondrion-associated endoplasmic reticulum membrane, Glycogen synthase kinase-3 β

## Abstract

While several hypotheses have been proposed to explain the underlying mechanisms of Alzheimer's disease, none have been entirely satisfactory. Both genetic and non-genetic risk factors, such as infections, metabolic disorders and psychological stress, contribute to this debilitating disease. Multiple lines of evidence indicate that ceramides may be central to the pathogenesis of Alzheimer’s disease. Tumor necrosis factor-α, saturated fatty acids and cortisol elevate the brain levels of ceramides, while genetic risk factors, such as mutations in *APP*, *presenilin*, *TREM2* and *APOE* ε4, also elevate ceramide synthesis. Importantly, ceramides displace sphingomyelin and cholesterol from lipid raft-like membrane patches that connect the endoplasmic reticulum and mitochondria, disturbing mitochondrial oxidative phosphorylation and energy production. As a consequence, the flattening of lipid rafts alters the function of γ-secretase, leading to increased production of Aβ_42_. Moreover, ceramides inhibit the insulin-signaling cascade via at least three mechanisms, resulting in the activation of glycogen synthase kinase-3 β. Activation of this kinase has multiple consequences, as it further deteriorates insulin resistance, promotes the transcription of *BACE1*, causes hyperphosphorylation of tau and inhibits the transcription factor Nrf2. Functional Nrf2 prevents apoptosis, mediates anti-inflammatory activity and improves blood–brain barrier function. Thus, various seemingly unrelated Alzheimer’s disease risk factors converge on ceramide production, whereas the elevated levels of ceramides give rise to the well-known pathological features of Alzheimer’s disease. Understanding and targeting these mechanisms may provide a promising foundation for the development of novel preventive and therapeutic strategies.

## Introduction

### A Pathology of Unanswered Questions

Mutations in the genes encoding amyloid precursor protein (*APP*), β-site *APP* cleaving enzyme-1 (*BACE1*) and *presenilin* are associated with early onset Alzheimer’s disease (AD), which is characterized by cognitive deterioration with histopathological findings such as extracellular amyloid-β (Aβ) plaques and intracellular tau neurofibrillary tangles (for summaries see [1, 2]). The amyloid cascade hypothesis, which has dominated the field for over 30 years, predicts that amyloid plaques cause the characteristic cognitive decline. However, the link between Aβ-accumulation and cognitive symptoms of AD may not be direct, since diffuse deposits of Aβ have been described in *post mortem* studies of patients who were cognitively unimpaired [3, 4]. In the same vein, amyloid positron emission tomography (PET) studies indicate that Aβ can be present in cognitively unimpaired elderly individuals [5]. Some patients with AD display tau-tangles, but lack plaques [6]. Further results casting doubt on the causal relationship between Aβ-accumulation and cognitive symptoms come from studies aiming to treat AD by reducing Aβ (γ-secretase inhibition, *BACE1* inhibition, amyloid antibody-therapy and passive immunization) failing to induce meaningful reductions in the pace of cognitive decline [5, 7, 8].

These findings suggest that Aβ deposits are not causally related to the cognitive deterioration of patients with AD [4, 9]. If plaques and cognition are not causally linked, then they might be parallel consequences of a common causal process [4, 7]. This, of course, immediately demands the identification of their potential common cause. There is an extensive list of factors that have been reported to be associated with or to contribute to AD, including inflammation, infection, central diabetes, hypotension, hypoperfusion, cholesterol, angiotensin II, oxidative damage, iron deregulation, cholesterol metabolism, blood–brain barrier (BBB) dysfunction and synuclein toxicity [1, 5, 7, 10, 11].

In this review, it is argued that many of these factors enhance ceramide accumulation in the brain. Ceramide overload can explain the formation of plaques, neuronal loss, tau-phosphorylation and central insulin resistance. These symptoms occur in parallel via different pathological processes, and each of these can be influenced by additional factors, such that each may vary in severity.

## Key Findings: Overview and Discussion

### Ceramides

**Fig. 1:**
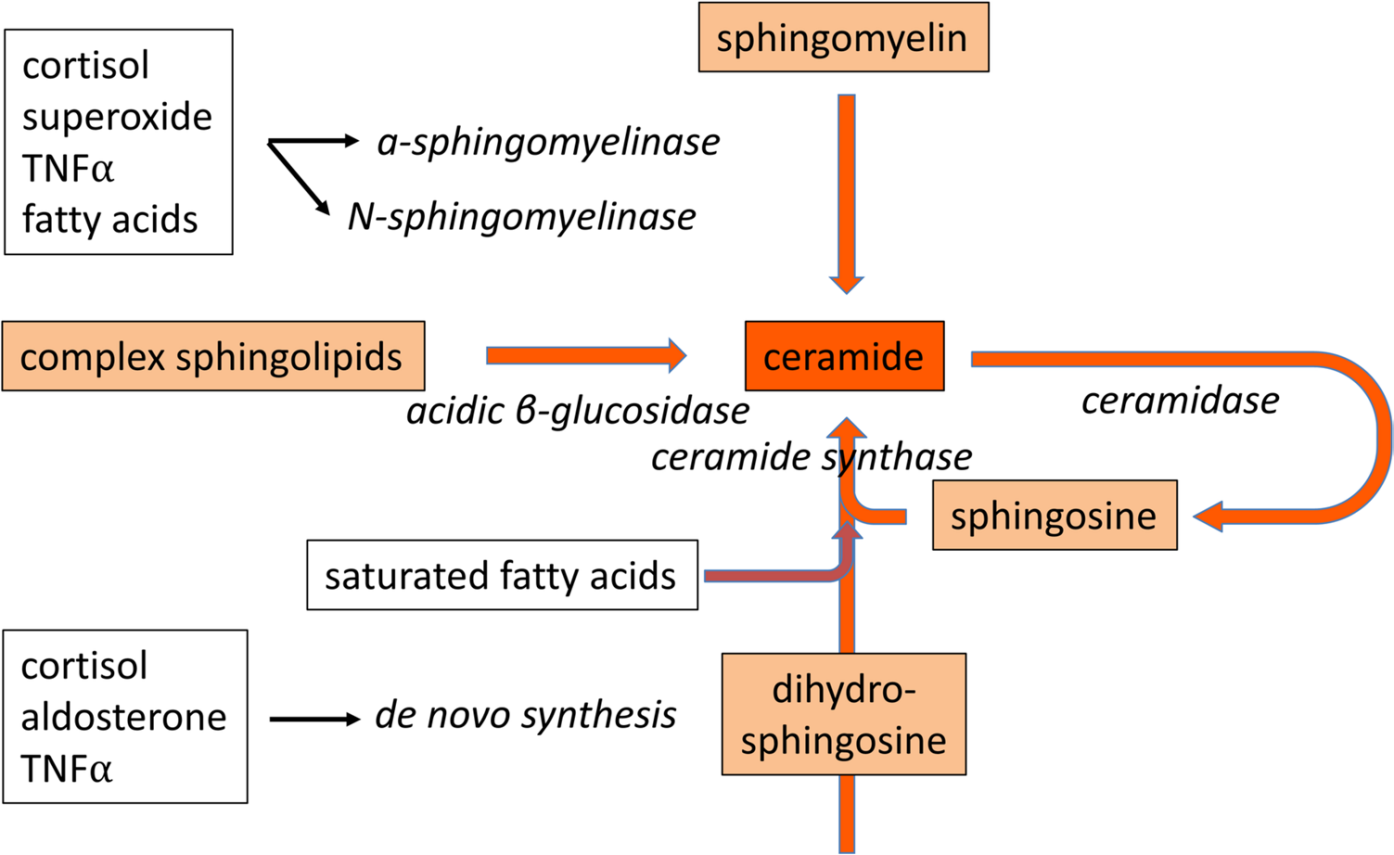
Ceramide levels are determined by the de novo synthesis pathway and by hydrolysis of sphingomyelin. Both are increased by various factors, such as cortisol (stress), pro-inflammatory cytokines (inflammation) and reactive oxygen species (ROS; oxidative stress), while de novo ceramide synthesis is induced by a diet high in saturated fatty acids. In addition, ceramide is produced as an intermediate in the “salvage pathway,” where complex sphingolipids such as gangliosides are metabolized into sphingosine, which can either be degraded or reused for the re-synthesis of sphingolipids. Abbreviation: TNFα, tumor necrosis factor-α

Ceramides are lipids that are composed of saturated fatty acids, with acyl-chain lengths of C_16_ to C_22_ (and occasionally longer forms), that are attached to an amine, which is generally sphingosine [12, 13]. Ceramides are enriched in both the plasma membrane and intracellular organelles, where they play multifaceted roles in signaling, cellular organization and responses to external stimuli [14]. Perturbations in ceramide levels can significantly impact various cell types across multiple biological contexts, resulting in diverse effects such as apoptosis, impaired cellular function and altered metabolism [15]. Recent studies, enabled by improvements in analytical methods for the detection and quantification of lipid molecules, have drawn attention to the presence of elevated levels of ceramides in blood [16, 17], cerebrospinal fluid (CSF) [18, 19], brain tissue [20–23] and the vicinity of plaques [10] of individuals with AD. It seems that ceramide accumulation generally occurs early in the disease course [20]. Ceramide levels are determined by several pathways [24, 25], two of which seem to be particularly relevant to AD. First, ceramides are generated de novo from serine and palmitoyl-CoA (yielding dh-sphingosine), followed by amide formation between dh-sphingosine and a fatty acid of variable acyl-length [26]. This latter step involves one of six iso-enzymes of ceramide-synthase, each of which preferentially functions with a specific acyl-chain length [26]. These ceramides are used to generate the membrane constituent sphingomyelin, involving the enzyme sphingomyelin-synthase (see Fig. 1). Sphingomyelin can be converted back to ceramide by a number of enzymes, collectively referred to as sphingomyelinases (SMase) [25]. This reversal step is the second major determinant of circulating ceramide levels. Additionally, ceramide levels are influenced by the “salvage pathway,” which involves the degradation of complex sphingolipids into ceramide, followed by further breakdown into sphingosine by ceramidases [27]. Sphingolipid metabolism in these three interrelated biosynthetic pathways—the de novo synthesis pathway, the sphingomyelin pathway and the salvage pathway—is regulated by more than 30 enzymes, each playing a distinct role in the synthesis, degradation and interconversion of sphingolipids (reviewed in detail in [27, 28]). Genetic mutations and enzymatic dysfunction of ceramidases can, in principle, lead to the accumulation of ceramides. In a microarray study in subjects with AD of various degrees of severity, the expression levels of the enzymes involved in ceramide synthesis were elevated and correlated with the progression of clinical dementia [29]. Furthermore, the cited study reported a reduced expression of the ceramidase enzyme ASAH1 in mild and severe AD.

### Mutations in APP, BACE1 and Presenilin Activate Neutral Sphingomyelinase

The original articulation of the amyloid cascade theory assumed that the C-terminal portion of APP is the cause of the cognitive decline in AD [30]. Cleavage of trans-membranal *APP* by *BACE1* generates two proteins: the C-terminal transmembrane peptide C99 and the soluble N-terminal fragment. Further cleavage of C99 by γ-secretase again produces two intracellular fragments: Aβ and the *APP* intracellular domain [31]. Since the dominant pathogenic role of extracellular Aβ has become progressively more doubtful [2, 8], the attention of researchers has shifted to the intracellular *APP* products. One subsequent hypothesis highlights the role of intracellular Aβ [8, 32–35], while another suggests that C99 is the causal factor for cognitive deterioration [2, 31]. These hypotheses are not mutually exclusive, since C99 still contains the Aβ peptide. Both hypotheses also highlight the role of the ER and its direct contact with mitochondria via the so-called mitochondria-associated ER-membrane (MAM) [2, 8]. An increase of C99 in MAM upregulates the production of phosphatidyl-serine [31], which is thought to result from enhanced function of the enzyme phosphatidyl-serine synthetase that is located in the MAM [36]. Phosphatidyl-serine is known to stimulate the activity of neutral sphingomyelinase (nSMase) [37, 38]. The nSMase2 isoform is activated by various cellular stressors, including IL- 1β, tumor necrosis factor-α (TNFα) and certain anti-tumor agents [39]. TNFα specifically triggers nSMase2 activation via a p38 MAPK-dependent pathway [40], a process that is reversed by the phosphatase calcineurin. However, oxidative stress inhibits calcineurin activity, thereby potentiating the TNFα-induced activation of nSMase2 [39]. As a result, both inflammatory conditions and hypoxic stressors—such as ischemia/reperfusion injury or exposure to cigarette smoke—can upregulate nSMase2 activity, leading to increased ceramide production [39]. This mechanism creates a feedback loop that exacerbates inflammatory response [41]. Mutations in *APP*, *BACE1* and *presenilin* that increase the level of C99 could potentially increase the hydrolysis of sphingomyelin by nSMase and thus raise ceramide levels in the MAM.

### Genetic risk Factors for Late-Onset AD Increase Ceramide Synthesis

Allelic variation in *ApoE* is a major risk factor for late-onset AD [42–45]. Homozygous carriers of the ApoE ε4-allele exhibit abnormal Aβ levels in CSF, positive amyloid scans and earlier symptom onset than individuals homozygous for the ε3-allele [46, 47]. ApoE isoforms have distinct functions in regulating brain lipid transport, glucose metabolism, neuronal signaling, neuroinflammation and mitochondrial function [45, 46]. Carriers of the ε4-allele display higher fasting triglyceride levels, a trend also observed in samples from normolipidemic adults and children, diabetic and obese individuals and hyperlipidemic subjects [48–50]. Patients with the combined risk factors of type 2 diabetes (T2D) and ε4-allele carrier status had a higher risk of developing AD than expected from the sum of the individual risks [51, 52]. This is notable because T2D is associated with elevated blood levels of triglycerides [53, 54]. In a longitudinal study of cognitively normal individuals, the fasting triglyceride levels at midlife predicted the presence of Aβ and tau pathologies 20 years later [55]. In a mouse AD model with ApoE ε4 expression exclusively in the liver, the Aβ pathology in the brain was exacerbated. Such an effect was not observed in mice hepatically expressing ApoE ε3. This result therefore indicates that peripherally generated ApoE ε4 contributes to AD pathology in the brain [56]. Further studies in mice indicate that *APOE* ε4, compared to *APOE* ε3, increases the expression of multiple ceramide species in the cerebral cortex [57]. Similarly, peripheral injection of insulin elevated brain levels of the ceramides C_16:0_, C_20_, C_24_ and C_24:1_ and impaired brain mitochondrial function and oxygen consumption in mice expressing the ApoE ε4 allele [58]. Myriocin (an inhibitor of de novo ceramide biosynthesis) reversed ceramide accumulation in the animal brain and mitochondrial dysfunction [58], suggesting that this accumulation may be the consequence of peripheral disease mechanisms [58]. Besides myriocin, many other inhibitors of the sphingolipid pathway have been suggested, including fumonisin B1, CX- 4945 GT11 (C8-cyclopropenylceramide), scyphostatin and α-mangostin [59]; however, only a few have been tested in animal models. One of them is cycloserine, a potent inhibitor of serine palmitoyltransferase, which—​in a study using a transgenic mouse model (TgCRND8)—​reduced long-chain ceramide levels without evident toxic effects [60]. In addition, positive correlations were observed between cortical serine palmitoyltransferase, ceramide and Aβ_42_ levels. Inhibition of SMase activity through FIASMAs (functional inhibitors of acid sphingomyelinase) presents a compelling avenue for AD treatment [61]. However, their therapeutic potential may be limited by the complexity of ceramide regulation and the multiple pharmacological actions of these compounds. Additionally, the de novo synthesis of ceramides can be triggered by diets that are rich in palmitate and other long-chain saturated fatty acids [13, 62–65].

Mutations in triggering receptor expressed on myeloid cells-2 (*TREM2*) are associated with late-onset AD [66–68]. *TREM2* is expressed in dendritic cells, osteoclasts, tissue macrophages and microglia [69]. In late-stage AD, *TREM2* mutations may affect the ability of microglia to phagocytose extracellular Aβ deposits [69, 70]. However, *TREM2* also affects the lipid metabolism in both the central nervous system and the periphery [71]. In mice, a high-fat diet causes an increase in macrophage abundance in white adipose tissue and hypertrophy of adipocytes [72]. *TREM2* expression in these macrophages was linked with an anti-inflammatory phenotype and increases in both phagocytosis and the metabolism of fatty acids [73]. *TREM2* knockout aggravated the consequences of high-fat diets, including insulin resistance and hepatic steatosis [72]. In the absence of macrophages with intact *TREM2*, the lipid-fed adipocytes generated large amounts of C_18_ and C_20_ ceramides [72]. These ceramides not only induced adipocyte apoptosis, but relevant amounts of ceramides also reached the circulation. Metabolomic experiments found that *TREM2* deficiency with obesity-induced elevation of serum ceramide levels was correlated with the impairment of insulin sensitivity. Blockage of the de novo pathway of ceramide synthesis restored the hepatic insulin sensitivity of lipid-fed *TREM2*-knockout mice [72]. These data indicate that loss-of-function variants in *TREM2*, in conjunction with a lipid-rich diet, could give rise to elevated levels of ceramide in the circulatory system [71]. As with *APOE* ε4, this may have negative effects on neuronal function and survival.

### Non-Genetic Risk Factors for AD Raise Ceramide Levels

Lifestyle factors such as chronic overnutrition promote the development of insulin resistance, metabolic syndrome and T2D and are associated with high blood levels of triglycerides [53, 74, 75]. Systemic diabetes mellitus is well known to increase the risk of dementia [74, 76–78]. Similarly, meta-analyses have noted that obesity during the midlife period (another proxy for high triglyceride levels) is correlated with the incidence of dementia [79, 80], whereas, as mentioned above, elevated blood triglyceride levels during mid-life are associated with an increased risk of AD [55]. Since excess amounts of fatty acids become incorporated into ceramides, it is logical that the circulating levels of different long-chain ceramide species are elevated in patients with metabolic syndrome [65, 81, 82]. It is thought that these ceramides are causally involved, and furthermore, their plasma levels could represent disease markers for metabolic syndrome and associated disorders like coronary artery disease, cerebrovascular disease, stroke and cancer [26, 65, 83]. For instance, research indicates that plasma ceramide and triglyceride levels are closely linked to pro-inflammatory cytokines such as IL-6 and TNFα. These inflammatory markers likely contribute to peripheral insulin resistance in patients with T2D and coronary heart disease. Specifically, a study by Boon and colleagues [84] found that ceramides in low-density lipoprotein (LDL) are elevated in individuals with T2D and are associated with inflammation and skeletal muscle insulin resistance. Similarly, a study by Mello and colleagues [85] found that elevated ceramide concentrations were correlated with increased circulating levels of inflammatory cytokines. Additionally, a study by Turpin and colleagues [86] showed that ceramide synthesis is significantly elevated in the adipose tissue of obese individuals, with increases in ceramide levels correlating with weight gain and a decline in insulin sensitivity. Animal studies confirm the causal link between high-fat diets, ceramide production and AD pathology. For instance, high-fat diets promoted the development of Aβ-plaques, neurofibrillary tangles and cognitive impairment in rodents [87–89]. Triglycerides at the BBB are hydrolyzed by the endothelial enzyme lipoprotein-lipase [90], and the locally released saturated fatty acids increase endothelial ceramide production [38, 62, 63, 91]. Importantly, it was shown that circulating triglycerides were able to induce insulin resistance in the brain in animal experiments [91, 92]. These findings are relevant, because they indicate that disorders associated with high triglyceride levels in the circulatory system influence brain function and AD pathology, presumably via an increase in ceramide levels.

Infectious agents, such as *Herpes simplex* [93] and *Treponema* [94], as well as inflammatory diseases (such as psoriasis [95, 96] and chronic periodontitis [97, 98], have also been identified as risk factors for AD in epidemiological surveys (reviewed in [1, 99]). The connection between periodontitis and AD has been attributed to pathogenic bacteria like *Porphyromonas gingivalis*, inflammation and elevated cytokine levels [100–102]. Acute and chronic systemic inflammation, as indicated by increased serum TNFα, was associated with a doubling of the rate of cognitive decline during the ensuing six months in patients with AD [103]. In rats, the systemic application of the bacterial cell membrane component lipopolysaccharide (LPS) provoked a rapid increase in ceramide levels in muscle, plasma and brain samples [104, 105]. Ceramide levels were also elevated in the CSF of patients with multiple sclerosis and, notably, reached mitochondrially toxic levels [106]. Similar to long-chain saturated fatty acids, TNFα is known to enhance de novo ceramide synthesis [107–109]. Additionally, TNFα may activate sphingomyelin hydrolysis, though the isoform of the involved SMase has not been definitely established [83, 107, 110].

Many AD patients hyper-secrete the stress hormone cortisol, and its plasma levels seem to be correlated with cognitive impairment and neuronal atrophy [1]. Moreover, stressful events are reported to increase dementia risk [111]. In rats that were exposed to chronic mild stress, a subset of vulnerable animals developed anhedonia and cognitive deficits; additionally, the hippocampal levels of Aβ_40_, *BACE1* and phosphorylated tau were increased, while phosphorylation of Akt was diminished [112]. In a similar rat experiment, exposure to chronic unpredictable stress evoked a reduction in sphingomyelin and an increase in ceramide (indicative of SMase activation) in the hippocampus and frontal cortex that was correlated with blood levels of corticosterone [113]. In mechanistic studies in cell lines, the glucocorticoid receptor agonist dexamethasone gave rise to sphingomyelin hydrolysis and ceramide accumulation by acid sphingomyelinase (aSMase) [114, 115]. In another study in rats, it was observed that chronic stress and corticosterone-dosing induced hyper-phosphorylation of tau in the hippocampus and prefrontal cortex (PFC) and impaired hippocampus- and PFC-dependent learning [116]. Transgenic mice overexpressing aSMase displayed higher enzyme activity and ceramide production in the hippocampus that was paralleled by a decline in both neurogenesis and neuronal survival [117, 118]. These data indicate that stress-induced activation of the glucocorticoid receptor evokes an aSMase-mediated increase in ceramides and presumably consequential neuronal apoptosis, AD neuropathy and cognitive deterioration. Thus, aSMase has recently been proposed as a therapeutic target for the treatment of AD [119].

### Functional Consequences of Ceramide Increase in the Brain

**Fig. 2:**
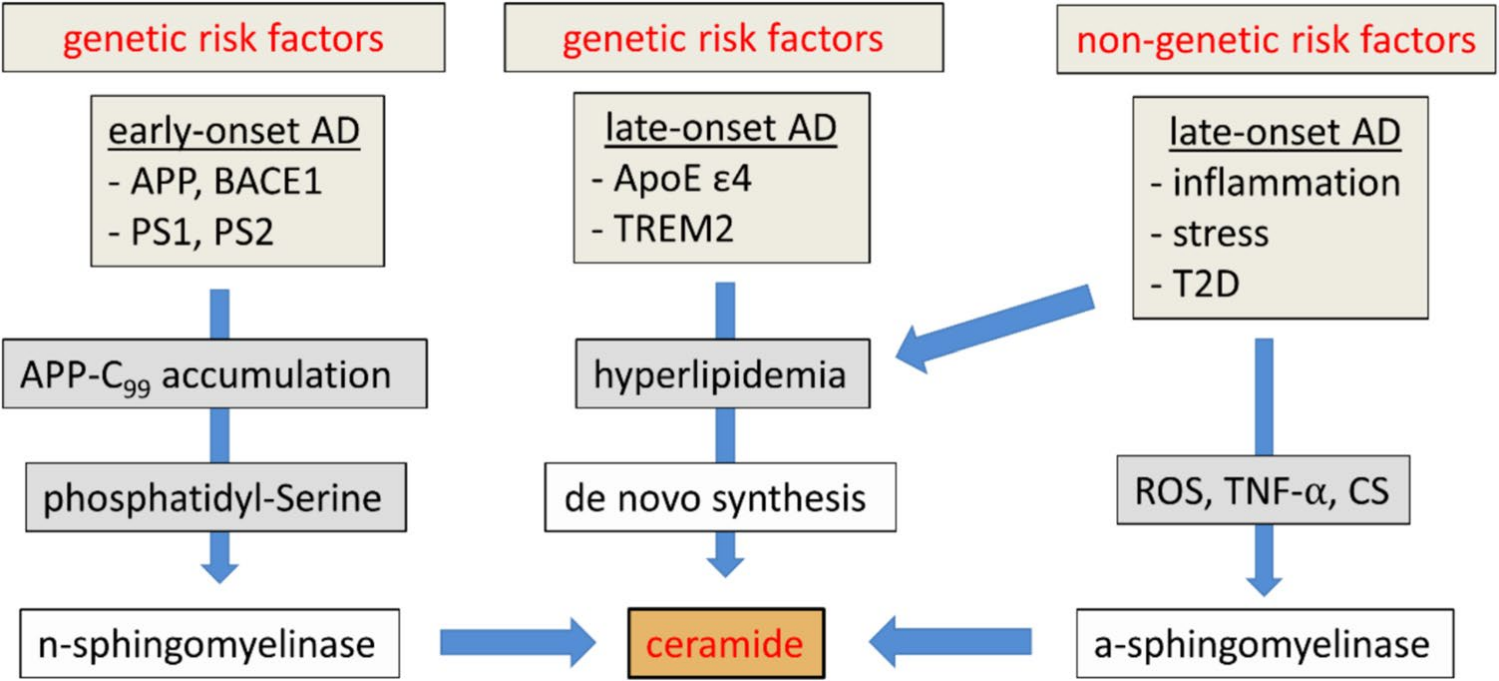
Summary of the pathways through which risk factors for AD converge on the elevation of ceramide levels. Abbreviations: AD, Alzheimer’s disease; ApoE ε4, apolipoprotein-E; APP, amyloid precursor protein; a-sphingomyelinase, acid sphingomyelinase; BACE1, β-site APP cleaving enzyme-1; CS, corticosterone; n-sphingomyelinase, neutral sphingomyelinase; PS1, presenilin-1; PS2, presenilin-2; ROS, reactive oxygen species; TNFα, tumor necrosis factor-α; TREM2, triggering receptor expressed on myeloid cells- 2; T2D, type 2 diabetes

In summary, risk factors for AD, including infections, metabolic disorder and stress, have been shown to induce the synthesis of ceramide via increases in TNFα, long-chain saturated fatty acids and cortisol, respectively (see Fig. 2). Other risk factors for AD, such as alcohol-induced steatohepatitis [120] or coronary artery disease [121], are similarly associated with peripheral synthesis and the accumulation of ceramides, whereas hemorrhagic stroke has been reported to elevate ceramide levels in CSF [122]. Preclinical experiments suggest that ceramide accelerates cellular senescence [25], which is noteworthy, since age is an obvious risk factor for AD [1]. Conversely, a reduction in the synthesis of ceramide improved the Aβ levels, plaque burden, tau hyperphosphorylation and cognitive function of transgenic mouse AD animal models [123]. This raises an important question: how could ceramides trigger brain insulin resistance, Aβ production, tau-phosphorylation and cognitive decline, the pathologies that define AD?

### AD and Central Insulin Resistance

Ceramides activate a number of signaling pathways related to insulin resistance. For example, ceramides activate c-Jun N-terminal kinase (JNK), although there is no agreement on the precisely elements of the signaling pathway [124–126]. JNK is also activated by inflammatory cytokines and Toll-like receptor 4 and 9 (TLR4 and TLR9) [127]. This enzyme produces inhibitory phosphorylation of insulin-responsive substrate (IRS) [127, 128]. Inhibitory phosphorylation of IRS interrupts both the insulin-receptor–IRS1–phosphatidyl inositol 3-kinase (PI3 K) pathway and the insulin-like growth factor-1 (IGF1)–IRS2–PI3 K pathway and contributes to insulin-resistance in the brain [128–131], an early and common feature of AD [126, 132, 133]. Inhibitory phosphorylation of IRS1 was positively correlated with the presence of oligomeric Aβ plaques, but was negatively associated with episodic and working memory performance [130]. It is thought that extracellular oligomeric Aβ serves as a further trigger for JNK activation and thus could lead to further IRS1-inhibition [126, 134]. This explanation, however, requires extracellular immunogenic Aβ, which may occur only relatively late in the disease course. Notably, a longitudinal study in a mouse model of AD indicated that JNK activation occurs much earlier in the disease course, when synaptic impairment had started to diminish responses in mice subjected to a radial arm water maze [135].

**Fig. 3:**
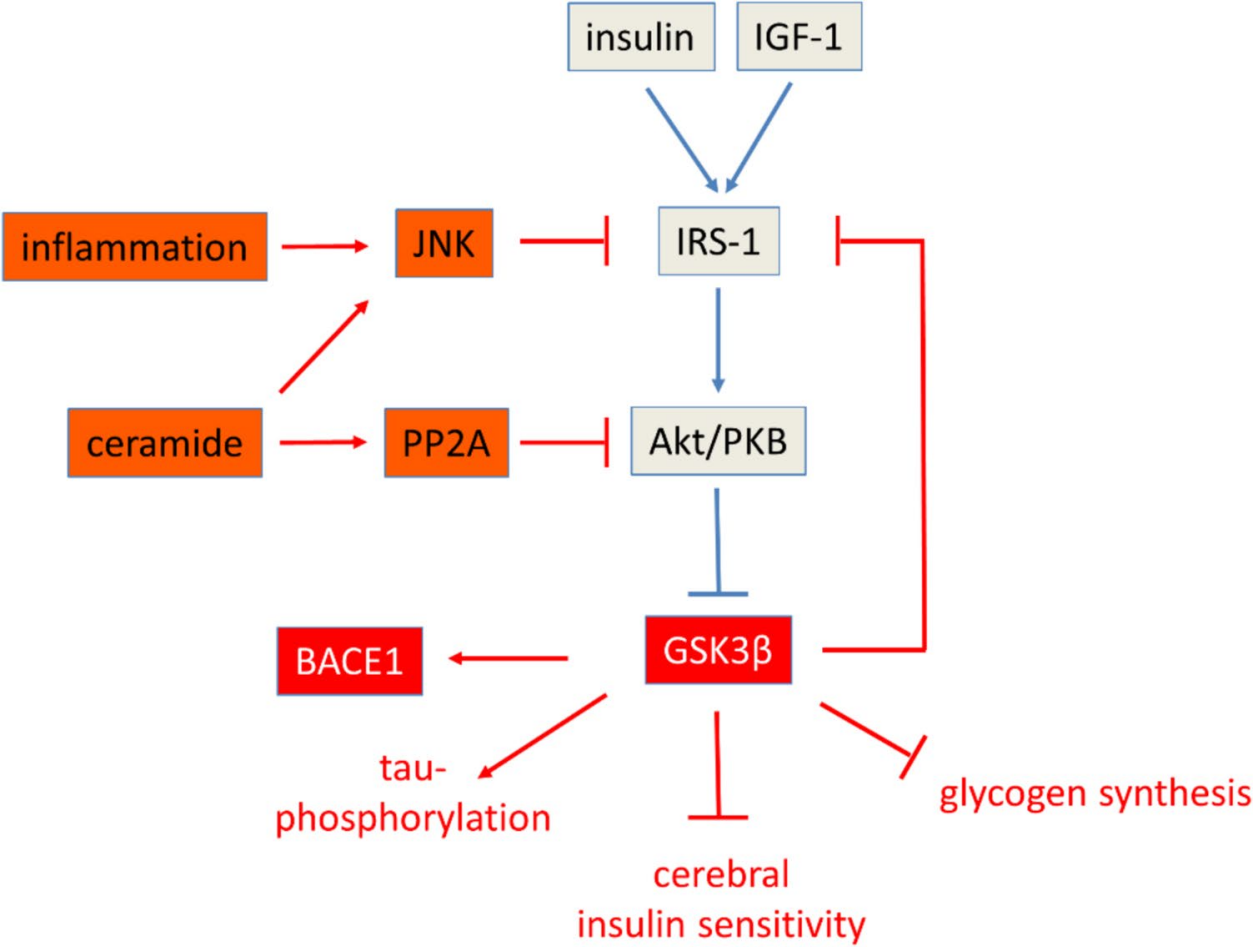
Insulin and insulin-like growth factor (IGF-1) activate a pathway that leads to inhibition of the kinase GSK3β. Ceramide activates PP2 A and JNK, both of which suppress the insulin/IGF-1 pathway. Active GSK3β has a number of consequences, including increased inhibition of the insulin pathway, increased transcription of BACE1, hyperphosphorylation of tau, glycogen synthesis and induction of insulin resistance in the brain. Abbreviations: Akt/PKB, protein kinase B; BACE1, β-site APP cleaving enzyme-1; GSK3β, glycogen synthase kinase-3 β; IGF, insulin-like growth factor-1; IRS-1, insulin receptor substrate-1; JNK, c-Jun N-terminal kinase; PP2 A, protein phosphatase-2 A

There are two more potential mechanisms for central insulin-resistance that involve ceramide. The first mechanism involves ceramide-induced activation of the atypical protein kinase C (PKC) isotype PKCζ, which inhibits PI3 K-Akt [136–138]. The second mechanism involves ceramide-induced activation of protein phosphatase-2 A (PP2 A), which dephosphorylates/inhibits Akt [139–142]. Activated by PI3 K, Akt is a major inhibitor of glycogen synthase kinase-3 β (GSK3β) [143, 144]. Thus, insulin signaling activates the PI3 K–Akt pathway, which inhibits GSK3β. An elevated level of ceramide, via the above-mentioned mechanisms, inhibits PI3 K and Akt, and thus activates GSK3β. Similar to JNK (see above), GSK3β is known to phosphorylate/inhibit IRS1 and thus suppresses insulin signal transduction as well [145, 146]. This process is summarized in Fig. 3.

### GSK3β Activity and BACE1 Expression

Brain glucose hypometabolism can precede the obvious clinical manifestations of AD by years [147, 148]. The combination of impaired insulin signaling, reduced Akt activity and increased GSK3β activity has been observed in the hippocampus and frontal cortex in association with human neurological disorders [148], as well as in aged mice [149]. Several aspects of AD pathology, including tau-hyperphosphorylation, apoptotic neuronal death, reactive astrocytosis and spatial learning deficits were recapitulated in a transgenic mouse strain with conditional overexpression of GSK3β specifically in the forebrain [150, 151] (reviewed in [152]). GSK3β is one of the enzymes responsible for the phosphorylation of tau [153–155], while GSK3β has been shown to accumulate in pre-tangle neurons [150]. Notably, active GSK3β promoted nuclear factor-κB (NFκB)-mediated *BACE1* transcription [152, 156, 157], and therefore, it is likely that it contributes to Aβ-production [150, 158, 159]. BACE1 expression and its enzymatic activity were elevated in temporal cortex autopsy samples from patients with sporadic AD [160]. BACE1 expression was also increased at the BBB in a mouse model of AD [161]. Increased BACE1 activity can be detected even in serum and thus could represent a suitable early biomarker for AD [162].

Nuclear factor erythroid 2-related factor 2 (Nrf2) is a transcription factor with multiple functions. It increases the transcription of genes with an antioxidant function, inhibits microglia-mediated inflammation and promotes an increase in the number of mitochondria [163, 164]. Interestingly, insufficient Nrf2-activation in humans has been linked to chronic diseases, including AD [164, 165]. In mice, the targeted disruption of the *Nrf2* gene enhanced inflammatory responses and increased intracellular APP and Aβ levels [166] and exacerbated the cognitive deficits of transgenic AD mice [167]. Conversely, Nrf2 activation suppressed Aβ-induced neuronal toxicity in vivo [168]. *Nrf2* inhibits *BACE1* expression by binding to so-called antioxidant response elements (AREs) in the *BACE1*-promotor region in AD animal models [169]. This response was accompanied by a reduction in Aβ-production and fewer cognitive deficits [169]. Importantly, active GSK3β promoted metabolism of Nrf2, consequently reducing Nrf2 transcriptional activity [163].

These data provide additional support for the proposal that inactivation of GSK3β could be a useful strategy for the treatment of AD [170], which is also supported by data from a genetic study. A single nucleotide polymorphism in the GSK3β gene (− 50 C/T SNP [rs334558]) influences the transcription of *GSK3β*, with the C-allele being the less active form [171]. As anticipated, the C/C homozygous genotype seems to protect against AD [154, 172]. In line with the model presented in Fig. 3, JNK activation also gives rise to apoptotic activity [127], and consequently, the inhibition of the JNK-signaling cascade has also been proposed as therapeutic target for AD [135].

### GSK3 Activity and Tau Phosphorylation

Similar to IRS1 (see above), the protein Tau—an integral component of neurofibrillary tangles—is phosphorylated at multiple sites by both GSK3 (α and β) and JNK [125, 150, 155]. Insults such as a high-fat diet, which induces brain inflammation and simultaneous activation of JNK and GSK3 [87], lead to tau hyperphosphorylation and cognitive dysfunction in preclinical models of AD [88, 125]. Similarly, chronic stress induced by olfactory bulbectomy activates GSK3 and JNK, resulting in tau hyperphosphorylation and memory deficits [173]. In animal studies, the non-ATP-competitive GSK3 inhibitor tideglusib reduced tau phosphorylation, Aβ deposition, astrocyte proliferation and ameliorated certain memory deficits. However, in two small phase II clinical trials, the compound failed to produce significant clinical improvements [152]. This outcome may suggest that GSK3 inhibition alone is insufficient—perhaps due to persistent JNK activity—or that tau hyperphosphorylation is not the primary driver of cognitive decline. In this context, it is also worth noting that the tau aggregation inhibitor LMTM failed in a phase III trial [6].

### Effect of Ceramides on MAM-Thickness and γ-Secretase Function

Proteolytic processing of *APP* by *BACE1* generates the C-terminal fragment C99. Thus, BACE1 hyperactivity is expected to result in elevated levels of C99. This indeed is observed not only in carriers of APP mutations, but also in patients with sporadic (i.e., non-familial) AD. Elevated levels of C99 can be monitored in platelets [174]. C99 is processed by γ-secretase, which is a molecular complex consisting of presenilin (two isoforms), nicastrin, anterior pharynx defective 1 (*APH-1*) and presenilin enhancer 2 (*PEN-2*) [175]. Presenilin-1 (PS1), presenilin-2 (PS2) and γ-secretase activity are predominantly located in specialized lipid raft-like membrane-contact points between the ER and mitochondria (MAMs) [176]. Aβ is produced in MAMs [177]. MAMs are also important for cholesterol and phospholipid lipid metabolism, calcium homeostasis and mitochondrial function [176]. The function of MAMs is altered in AD [31, 178]. Presenilins and GSK3β physically interact, and the non-selective GSK3 inhibitor lithium has been shown to block Aβ-production by interfering with C99-cleavage at the γ-secretase step [158]. Since ceramides activate GSK3β, the expected consequence of ceramide accumulation would be increased γ-secretase-mediated cleavage of C99. Such an effect has been observed in a study that utilized synthetic ceramide analogues [179]. These authors reported that the ceramide analogues specifically increased production of Aβ_42_, but not Aβ_40_ [179]. Whether a similar effect could be achieved by endogenous ceramides was not investigated, unfortunately. An alternative explanation for preferential Aβ_42_ production is provided by Area-Gomez and Schon [2]. Since in AD the MAM-raft is thinner than usual, they hypothesized that the γ-secretase complex is somewhat tilted to fit within the thinner lipid raft, thus leading to the cleavage of C99 at a slightly different position [2]. As ceramides affect the localization of cholesterol and the rigid lipid-order within the rafts [180, 181], it is reasonable to assume that the thinner diameter of the lipid raft is a direct consequence of elevated ceramide accumulation. Thus, the hypothesis posited by Area-Gomez and Schon [2] is compatible with our hypothesis regarding the role of elevated ceramide levels in AD.

However, it is unclear if the elevated synthesis of either Aβ_40_ or Aβ_42_ is relevant to the early memory loss associated with AD. In a study on mice carrying both PS1 and Swedish β-APP mutations, a marked age-dependent increase in cortical and hippocampal Aβ deposition was found, but this change was insufficient to cause the dramatic neuronal loss in the hippocampus and the association cortex that is characteristic of AD [182]. This is consistent with the unsatisfactory efficacy of clinical approaches that target a reduction in Aβ.

### Apoptosis Owing to Mitochondrial Dysfunction

Both active GSK3β and inactive Nrf2 have negative effects on mitochondrial biogenesis, mitochondrial motility, mitochondrial permeability and mitochondria-dependent apoptosis [168, 170, 183]. Nrf2 activates peroxisome proliferator-activated receptor γ coactivator 1α (*PGC1α*) and nuclear respiratory factor-1 (Nrf1), two factors that stimulate mitochondrial biogenesis [184]. Furthermore, Nrf2 inhibits oxidative stress-mediated opening of the mitochondrial permeability transition pore, counterbalances mitochondrial reactive oxygen species (ROS) production, reduces the release of mitochondria-derived toxins and regulates mitochondrial quality control among its other basic functions [184, 185]. There are Nrf2-responsive ARE sequences in the promotors of nuclear and mitochondrial genes coding for elements of the mitochondrial oxidative phosphorylation machinery, such that Nrf2 hypofunction causes diminished adenosine-triphosphate (ATP) production [185]. Mitochondrial dysfunction is hypothesized to be a major driver of the histopathologic and pathophysiologic features of late-onset sporadic AD [126, 148, 186, 187]. Nrf2 levels in hippocampal samples from AD patients were significantly lower than those in samples from controls [169, 188], and they were inversely correlated with Aβ deposition [169]. Moreover, the transcriptional activity of Nrf2 seems to decline with age [189, 190]. Notably, both longevity [191] and AD pathology [192] display maternal overrepresentation, suggestive of mitochondrial inheritance playing a role in AD [186].

### Effects of Ceramides on Mitochondrial Function Independent of GSK3β

High ceramide levels induce apoptosis and cytotoxicity in a wide variety of cells, including neurons [106]. This apoptotic response specifically involves ceramide production in mitochondria and can be rescued by overexpression of B-cell lymphoma- 2 (BCL2) [193]. The activity of BCL2 depends on phosphorylation at Ser-70. Ceramide-induced PP2 A activity in mitochondria leads to dephosphorylation of BCL2, which contributes to ceramide-induced apoptotic cell death [194]. Ceramide levels in mitochondria are mainly determined by the activity of aSMase and by de novo synthesis. Mitochondrial ceramide accumulation occurs after inflammation (TNFα), administration of chemotherapeutic drugs or corticosteroids, ischemia/reperfusion, Fas antigen activation, irradiation or treatment with ROS [193]. Mignard and colleagues investigated the ceramide metabolic pathway both in vivo and in vitro in purified ER, MAMs, and mitochondria in mouse liver, mouse brain, and a human glioma cell line in response to staurosporine-induced apoptosis. Ceramide (especially C_16_-ceramide) levels increased during early apoptosis, through hydrolysis of sphingomyelin by aSMase in the MAM and its subsequent transfer to mitochondria [195]. Ceramide species with different acyl-chain lengths display distinct biophysical properties and are associated with decreased mitochondrial respiratory chain activity, increased ROS production and oxidative stress, mitochondrial outer membrane permeabilization, reduced mitochondrial membrane potential, mitophagy and apoptosis [196]. The de novo synthesis of ceramides involves ceramide synthases (CerS), a group of enzymes that catalyze the formation of ceramides from sphingosine and acyl-CoA substrates. Six mammalian CerSs (CerS1–CerS6) have been identified and are enriched in the ER and mitochondria [197]. The expression of ceramide synthase is elevated by inflammatory signals, while the resulting ceramides subsequently impair mitochondrial respiration [198–200]. While C_16_-ceramide is mainly synthesized by CerS5 and CerS6 [197] and stimulates cell apoptosis, it has a limited effect on systemic insulin-resistance [82]. Moreover, C_16_-ceramide directly inhibits complex IV, but the exact mechanism by which ceramides inhibit electron transport is still incompletely understood [196, 201]. In addition, ceramides with different acyl-chain lengths form large stable pores with deleterious effects on mitochondrial membrane polarization and permeabilization [196, 202]. BCL2-associated X (BAX) is recruited to ceramide-rich mitochondrial membranes, which results in an elevation of membrane permeability, an effect that is abrogated by BCL2 [196]. Additionally, C_18_-ceramide, but not C_16_-ceramide, in the outer mitochondrial membrane activates autophagic removal of mitochondria [196]. With respect to skeletal muscle and brain tissue, CerS1 and CerS4 are the most important isotypes. These enzymes preferentially produce C_18_-ceramide and C_20_-ceramide. Many details of the specific effects of ceramides with different acyl-chain lengths remain to be discovered; however, it can generally be concluded that ceramide accumulation in mitochondria results in their dysfunction, with negative consequences on oxidative capacity, controlled oxidative stress, undisturbed respiratory chain function, ATP production and mitochondrial proliferation [13, 138, 200], effects that correspond to fundamental aspects of AD [58].

### Mitochondrial Dysfunction as a Cause of Synapse and Neuron Loss

The examples detailed above demonstrate that ceramides either directly or indirectly, via *GSK3β* and *NRF2*, disturb mitochondrial function. The immediate consequence of that disturbance is a decline in energy production. Trafficking of mitochondria from the perinuclear area to the synapse is a process that requires energy, and dysfunctional mitochondria may therefore not reach the synapse [187, 200]. The loss of functional synapses is a plausible mechanism explaining the early decline in cognitive function and learning deficits observed in AD [203]. In agreement, glucose utilization in the brain is reduced prior to dementia onset [147]. An essential role of mitochondrial dysfunction in AD was already formulated two decades ago, as described in the mitochondrial cascade hypothesis posited by Swerdlow & Khan [186]. They suggested that oxidative damage to mitochondrial DNA, RNA, lipids and proteins is the starting point for the initiation of a vicious cycle, ultimately leading to a loss of neurons and their insufficient replacement by neurogenic progenitors [186, 204]. Neurogenesis is another process that requires a high amount of energy, and ceramide-induced mitochondrial dysfunction would be expected to lead to a gradual loss in neuronal progenitor cells [205]. Indeed, disturbed oxidative phosphorylation and reduced ATP production impair neurogenesis and neural plasticity [206]. Neurogenesis takes place in the dentate gyrus and the olfactory bulb. Atrophy and volume reductions in the hippocampal left pre-subiculum [207–209] and the right CA1 region [209, 210] have been described in early predementia and were found to be correlated with performance on memory tests. It seems that the cognitive decline is at least partly independent of the occurrence of amyloid plaques or tau-tangles [208, 211]. Notably, the cognitive impairment observed in patients with T2D is also associated with atrophy of the left pre-subiculum and right CA1 area [212]. However, this was not observed in T2D patients without cognitive decline [212]. In addition, neurogenesis and cognitive function were severely disturbed in an animal model of AD [213]. Olfactory dysfunction is another early symptom and potential biomarker for AD [214, 215] that seems to be unrelated to the Aβ burden [216].

### The Role of Triglycerides, Lipoproteins and Ceramides in the BBB Dysfunction

Triglycerides are produced by adipose and liver tissue and are transported after packaging into lipoproteins. When these lipoproteins reach their target tissue, they are catabolized by lipoprotein lipase (LPL), which induces the release of free fatty acids. The fatty acids bind to lipoprotein receptors at the cell membrane and are transported into the cell, where they can be incorporated in ceramides [90]. Importantly, LDL particles also contain sphingomyelin and ceramide [217]. SMase (from endothelial cells) can convert sphingomyelin into ceramide [217, 218]. Systemic inflammation activates SMase and can thus increase the ceramide levels of circulating LDL particles [219]. LDL binds to the LDL-receptor on endothelial cells, whereafter the ceramide and sphingomyelin content is transferred to these cells. Following ceramide uptake, part of the LDL-derived ceramide accumulates inside the cells and so contributes to atherosclerosis [217], vascular remodeling [218] and endothelial apoptosis [220]. Oxidative stress (caused by ROS) and inflammatory cytokines like TNFα activate nSMase activity and further enhance the accumulation of LDL-derived ceramide and the rate of apoptosis [220, 221]. Apoptosis of pericytes and microvascular endothelium of the BBB could explain the functional decrease in barrier function that has been reported in the early stages of AD [222–224], especially in *APOE* ε4 carriers [225]. Notably, the tight junction molecule claudin-5 [226], which is involved in the dysfunction of the BBB in AD [224], is a target gene of Nrf2 [227]. Increasing Nrf2 function rescues the loss of claudin-5 and improves the BBB function [227]. Thus, it can be hypothesized that ceramides contribute to the early dysfunction of the BBB in AD.

BBB dysfunction is an early feature of AD, occurring well before the onset of dementia [222–224]. This dysfunction involves both morphological alterations in cellular components—such as endothelial cells, astrocytes and particularly pericytes [225]—as well as molecular changes, including increased BACE1 expression at the abluminal side of the microvascular endothelium [161], and reductions in tight junction proteins, membrane transporters and the basal lamina [224, 225] (Halliday et al., 2016; Alkhalifa et al., 2023). Choi et al. [119] argue that elevated levels of aSMase in the blood and brain of patients with AD lead to increased ceramide production in cells of the BBB. According to the authors, this may account for impaired cerebral blood flow autoregulation, neuronal death, abnormal autophagy, neuroinflammation, BBB disruption and altered BACE1 and Aβ production. Increased ceramide in endothelial cells also compromises mitochondrial function, with detrimental effects on BBB integrity [228]. Moreover, a functional endothelium is essential for glucose transport across the BBB—an activity known to be impaired in AD [229]. Finally, reduced viability of astrocytic mitochondria impairs the cells' ability to metabolize extracellular Aβ [230], potentially explaining the accumulation of Aβ in the perivascular space [161].

## Integrative Synthesis and Forward-Looking Insights

**Fig. 4:**
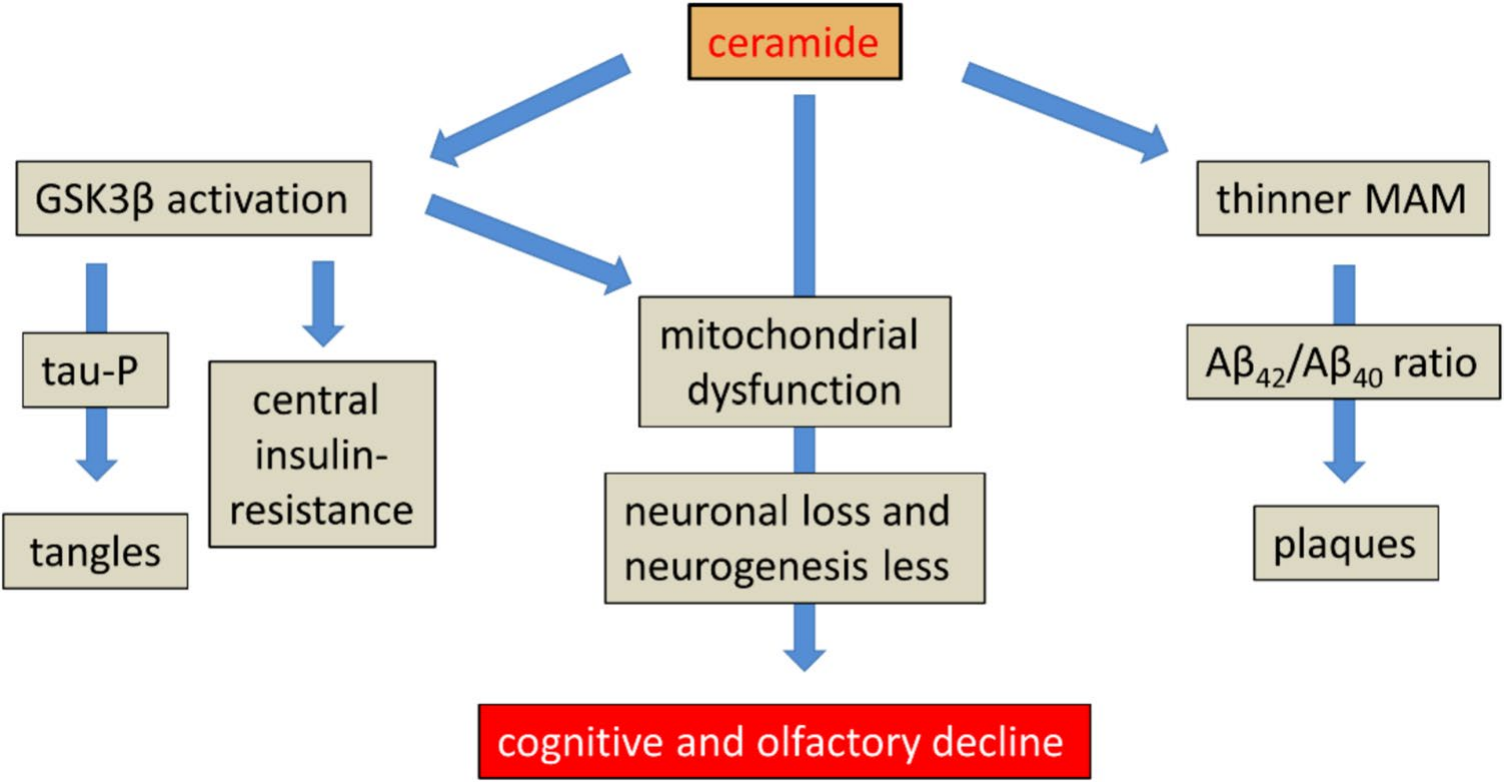
Ceramide accumulation in the brain is consistent with many of the pathological characteristics of Alzheimer’s disease. Abbreviations: GSK3β, glycogen synthase kinase-3 β; MAM, mitochondria-associated endoplasmic reticulum membrane

According to the hypothesis of ceramide involvement presented here, the neuronal accrual of ceramides is responsible for all the pathophysiological hallmarks of AD (see Fig. 4). This hypothesis, however, leaves unclear why tau tangles and extracellular Aβ plaques can occur without substantially affecting cognitive function. Cognitive dysfunction is thought to be the consequence of a loss in synapses and neurogenesis. Accordingly, one possible explanation is that these functions are spared despite increasing ceramide levels. In animal studies, physical exercise and environmental enrichment have been shown to promote neurogenesis [231, 232]. Similarly, in aging humans, the maintenance of physical and mental activity is known both to preserve cognitive performance and to reduce the risk of AD [233, 234].

However, one important problem remains unsolved. AD occurs much more frequently in females than in males [235]. We propose a speculative hypothesis for this discrepancy. Aldosterone, acting via the mineralocorticoid receptor (MR), has been reported to increase the expression of CerS1 and thus increase the de novo synthesis of C_18_-ceramide [236]. Notably, healthy females have higher circulating levels of aldosterone than males, and this difference becomes accentuated after the menopause [237]. Moreover, it has been reported that aldosterone levels are correlated with adiposity in females, but for unknown reasons, this is not observed in males [238, 239]. Apart from the adrenal cortex, mature adipocytes also express the enzyme aldosterone synthase (cytochrome P450 family 11 subfamily B member 2ER, CYP11B2) and release aldosterone [240], such that plasma aldosterone levels generally are higher in individuals with abdominal adiposity [241]. The expression of the MR is stimulated by progesterone [242], whereas estradiol increases CYP11B2 expression via stimulation of the orphan receptor G protein-coupled estrogen receptor 1 (GPER1) [237]. There is limited evidence that aldosterone promotes cognitive decline [243] and premature cell ageing [244]. Conversely, angiotensin II receptor type 1 (AT1R) antagonists, which reduce aldosterone production, have been reported to decrease the risk of AD [245, 246]. Although it remains to be investigated if aldosterone raises ceramide levels in humans, the findings described above suggest that aldosterone could be a factor that disproportionally raises the risk of AD in females. Documentation of this effect would imply that MR antagonists might be useful in reducing AD risk, particularly in women, but perhaps in men too.

Other prophylactic treatments are conceivable as well. The pathological progression might be interrupted at selected nodes of the signaling cascade, for instance, by blocking the enzymes that are involved in ceramide synthesis, by inhibition of GSK3β or by increasing the activity of Nrf2. To evaluate the efficacy of such treatments, one might consider measuring downstream effects of Nrf2, such as increases in antioxidant proteins [247] or in the expression of autophagy-effectors. It is possible that elevated levels of ceramide in blood and CSF could serve as a biomarker of dementia risk.

Ceramide elevation is driven by multiple factors (Fig. 2), and selective inhibition of either aSMase, nSMase or key steps in the de novo pathway may not offer a universally effective intervention. An alternative approach could be to enhance ceramide clearance. There are five distinct ceramidases, as well as two adiponectin receptors, that contribute to the degradation of ceramide into sphingosine [27, 248]. Ceramidases are classified based on the pH optimum of their catalytic activity into acid (ASAH1), neutral (ASAH2) and alkaline (ACER1, ACER2 and ACER3) subtypes. The adiponectin receptors, AdipoR1 and AdipoR2, are integral membrane proteins with a seven-transmembrane domain structure; however, the orientation of their amino acid sequence is reversed compared to that of classical G-protein-coupled receptors [249]. Members of the ACER family are structurally related to the adiponectin receptors and share a seven-transmembrane domain architecture with the same amino acid orientation [248, 250]. Anti-obesity treatments such as PPARγ agonists (‘glitazones’), HMG-CoA reductase inhibitors (‘statins’), glucagon-like peptide-1 (GLP-1) agonists, as well as fish oil (ω-3 fatty acids), have been shown to increase adiponectin levels [251–253]. These treatments also reduce ceramide production and plasma levels [254–258]. There is also evidence that each of these approaches may delay the onset and progression of dementia [259–262]. However, it should be noted that circulating adiponectin levels may not be suitable as a biomarker [263], as the pharmacological response to adiponectin depends on the presence of the co-receptor T-cadherin [264]. Interestingly, certain synthetic adiponectin mimetics act independently of T-cadherin; however, these compounds are not yet in clinical development. Based on the information cited above, ceramidase activation appears to be a promising strategy to reduce the risk of dementia. This is further reinforced by the observation that GLP-1 agonists can increase the expression of ACER2 [265].

The plasma levels of ceramides do not largely differ between young men and young women, although levels of dihydro-ceramides are higher in females [266, 267], suggesting greater rates of de novo ceramide synthesis in females. After menopause, women have higher plasma levels of all ceramides and dihydro-ceramides and exhibit a greater age-related increase, in particular for the ceramides C_16:0_, C_18:0_ and C_20:0_ [268]. Such a profile is consistent with the increased AD prevalence observed in females. An age-related increase in all ceramide species for both sexes was reported by Weir et al. [269], whereas measures predictive of pre-diabetes and diabetes, such as body mass index, waste-to-hip ratio and triglyceride levels, were strongly associated with levels of dihydro-ceramides and Cer_18:0_ [268, 269]. Again, such profiles are consistent with age and obesity being risk factors for AD. In conclusion, lipid profiles observed in large population cohorts are consistent with, and therefore indirectly support, our hypothesis that elevated levels of ceramides with lipid chain-lengths of C_16_–C_20_ might represent a key factor in AD pathogenesis.

## Data Availability

No datasets were generated or analysed for the current study.

## Abbreviations

AD: Alzheimer’s disease
APH-1: Anterior pharynx defective 1
APOE: Apolipoprotein E
APP: Amyloid precursor protein
ARE: Anti-oxidant response element
ASMase: Acid sphingomyelinase
ATP: Adenosine-triphosphate
AT1R: Angiotensin II receptor type 1
BACE1: β-Site APP cleaving enzyme-1
BAX: BCL2-associated X
BBB: Blood–brain barrier
BCL2: B-cell lymphoma-2
CerS: Ceramide synthase
CSF: Cerebrospinal fluid
CYP11B2: Cytochrome P450 family 11 subfamily B member 2
ER: Endoplasmic reticulum
GSK3β: Glycogen synthase kinase-3 β
GPER1: G protein-coupled estrogen receptor 1
IGF1: Insulin-like growth factor-1
IRS1: Insulin receptor substrate-1
JNK: C-Jun N-terminal kinase
LDL: Low-density lipoprotein
LPL: Lipoprotein lipase
LPS: Lipopolysaccharide
MAM: Mitochondria-associated endoplasmic reticulum membrane
MR: Mineralocorticoid receptor
NFκB: Nuclear factor-κB
Nrf2: Nuclear factor erythroid 2-related factor 2
NSMase: Neutral sphingomyelinase
PEN- 2: Presenilin enhancer 2
PET: Positron emission tomography
PFC: Prefrontal cortex
PGC1 α: Peroxisome proliferator-activated receptor γ coactivator 1α
PI3 K: Phosphatidyl-inositol 3-kinase
PKC: Protein kinase C
PP2 A: Protein phosphatase-2A
PS1: Presenilin-1
ROS: Reactive oxygen species
SMase: Sphingomyelinase
TLR4/TLR9: Toll-like receptor 4 / Toll-like receptor 9
TNFα: Tumor necrosis factor-α
TREM2: Triggering receptor expressed on myeloid cells-2
T2D: Type 2 diabetes
